# Optimization of collimator angles in dual-arc volumetric modulated arc therapy planning for whole-brain radiotherapy with hippocampus and inner ear sparing

**DOI:** 10.1038/s41598-021-98530-7

**Published:** 2021-09-24

**Authors:** Wuji Sun, Kunzhi Chen, Yu Li, Wenming Xia, Lihua Dong, Yinghua Shi, Chao Ge, Xu Yang, Libo Wang, Huidong Wang

**Affiliations:** 1 Department of Radiation Oncology and Therapy, The First Hospital of Jilin University, Changchun, 130021 China; 2 Jilin Provincial Key Laboratory of Radiation Oncology and Therapy, Department of Radiation Oncology and Therapy, The First Hospital of Jilin University, Changchun, 130021 China; 3 NHC Key Laboratory of Radiobiology, School of Public Health, Jilin University, Changchun, 130021 China

**Keywords:** Non-small-cell lung cancer, Radiotherapy, Techniques and instrumentation

## Abstract

To optimize the collimator angles in dual-arc volumetric modulated arc therapy (VMAT) plans for whole-brain radiotherapy with hippocampus and inner ear sparing (HIS-WBRT). Two sets of dual-arc VMAT plans were generated for 13 small-cell lung cancer patients: (1) The collimator angles of arcs 1 and 2 (*θ*_1_/*θ*_2_) were 350°/10°, 350°/30°, 350°/45°, 350°/60°, and 350°/80°, i.e., the intersection angle of *θ*_1_ and *θ*_2_ (Δ*θ*) increased. (2) *θ*_1_/*θ*_2_ were 280°/10°, 300°/30°, 315°/45°, 330°/60°, and 350°/80°, i.e., Δ*θ* = 90°. The conformity index (*CI*), homogeneity index (*HI*), monitor units (MUs), and dosimetric parameters of organs-at-risk were analyzed. Quality assurance for Δ*θ* = 90° plans was performed. With Δ*θ* increasing towards 90°, a significant improvement was observed for most parameters. In 350°/80° plans compared with 350°/10° ones, *CI* and *HI* were improved by 1.1% and 25.2%, respectively; MUs were reduced by 16.2%; minimum, maximum, and mean doses (*D*_100%_, *D*_max_, and *D*_mean_, respectively) to the hippocampus were reduced by 5.5%, 6.3%, and 5.4%, respectively; *D*_mean_ to the inner ear and eye were reduced by 0.7% and 5.1%, respectively. With Δ*θ* kept at 90°, the plan quality was not significantly affected by *θ*_1_/*θ*_2_ combinations. The gamma-index passing rates in 280°/10° and 350°/80° plans were relatively lower compared with the other Δ*θ* = 90° plans. Δ*θ* showed a significant effect on dual-arc VMAT plans for HIS-WBRT. With Δ*θ* approaching 90°, the plan quality exhibited a nearly continuous improvement, whereas with Δ*θ* = 90°, the effect of *θ*_1_/*θ*_2_ combination was insignificant.

## Introduction

Whole-brain radiotherapy (WBRT) has been used for the treatment of brain metastases or the prophylactic cranial irradiation of patients with small-cell lung cancer in the past decades^1–3^. However, the neurocognitive deficits caused by the radiation, including memory loss and cognitive impairment, have always been a concern^4,5^. The damage to the neural stem cell compartment in the hippocampus is generally believed to be the main cause^6,7^. The Radiation Therapy Oncology Group (RTOG) 0933 trial has demonstrated promising results in reducing the adverse neurocognitive effect of WBRT with hippocampus and inner ear sparing (HIS-WBRT) compared with conventional WBRT^7–9^. In the RTOG 0933 protocol, the target coverage and the dose to the organs-at-risk (OARs) were strictly constrained to achieve a reasonable dose distribution, and intensity-modulated radiation therapy (IMRT) modalities are required^9^. Volumetric modulated arc therapy (VMAT) is usually preferred because of its higher delivery efficiency than fixed-gantry IMRT^10,11^.

Current VMAT implementation does not allow collimator rotation during delivery, and the collimator angle in each arc is required to be determined before plan optimization^12^. Numerous studies have investigated the effect of collimator angle in many aspects, including target coverage, normal tissue sparing, monitor units (MUs), and delivery accuracy, and the optimal collimator angle varied for different target sites and modalities^12–23^. For the complex-shaped target volume in HIS-WBRT, an optimal setting for the collimator angles must be applied, which, to the knowledge of the authors, has yet to be investigated.

Apart from the specific angle of each arc, the intersection angle between arcs should be investigated to optimize the collimator angle. In particular, for dual-arc VMAT, the intersection angle between two collimator settings may have a significant effect on the plan optimization. By rotating the collimator between arcs, a better dose distribution could be achieved with the multi-leaf collimator (MLC) moving directions orthogonal to each other^24^.

In this study, the collimator angle of dual-arc VMAT for HIS-WBRT was optimized in two steps. First, the plan quality with different intersection angles between arcs was evaluated to find the most adequate choice. Second, with the optimal intersection angle, the specific collimator setting of each arc was investigated on the basis of plan quality evaluation and quality assurance (QA).

## Materials and methods

### Patient selection and contouring

A total of 13 patients with small-cell lung cancer, who had been previously treated in 2018–2020 in the First Hospital of Jilin University, were retrospectively included. The patients were immobilized in supine position by using a thermoplastic mask (Klarity Medical & Equipment Co. Ltd., Guangzhou, China). Computed tomography (CT) images of the entire head region were acquired using Philips Brilliance Big Bore CT scanner (Philips Healthcare, Cleveland, OH) with 3 mm slice thickness. The T1-weighted contrast-enhanced magnetic resonance imaging sequences, obtained with 1 mm slice thickness, were fused to the plan CT in the Eclipse version 13.5 (Varian Medical Systems, Palo Alto, CA) treatment planning system (TPS).

Anatomical contouring was carried out in accordance with the RTOG 0933 protocol^9^, including the whole brain, hippocampi, inner ears, lenses, optical nerves, and eyes. The planning target volume (PTV) for HIS-WBRT was defined as the whole brain with a 5 mm margin expansion excluding the hippocampus-sparing region. The hippocampus-sparing region was obtained by adding a 5 mm margin to the hippocampi, for the dose gradient between the hippocampus and PTV. The volumes (mean ± standard deviation (minimum and maximum)) of PTV, hippocampus, and inner ear were 1366.9 ± 114.3 (1099.3 and 1572.5), 3.7 ± 1.1 (1.0 and 5.2), and 1.3 ± 0.6 (0.2 and 2.4) cm^3^, respectively.

### Treatment planning

VMAT plans were optimized on Eclipse version 13.5 for 6 MV X-ray beams of a Varian TrueBeam linear accelerator equipped with a Millennium MLC-120 MLC (Varian Medical Systems, Palo Alto, CA). The maximum dose rate was 600 MUs/min. The dose distributions were calculated using the anisotropic analytic algorithm with a grid of 2.5 mm. The jaw tracking was enabled, and the maximum field size (X × Y) was 15 cm × 40 cm to adequately cover the whole brain.

For all patients, a dose of 30 Gy was prescribed in 10 fractions, and all plans complied with the dose criteria of the RTOG 0933 protocol and additional constraints used in this study, as shown in Table 1^9,25^. Dual-arc VMAT plans for HIS-WBRT were generated for each patient with two coplanar whole arcs. The settings of two collimator angles are denoted by *θ*_1_/*θ*_2_, i.e., the angles of the first and second arcs. The intersection angle between these two angles, denoted by Δ*θ*, ranged from 0° to 90°. Two sets of plans with different *θ*_1_/*θ*_2_ were generated as follows:

1. *θ*_1_/*θ*_2_ were set as 350°/10°, 350°/30°, 350°/45°, 350°/60°, and 350°/80°, i.e., Δ*θ* increased from 20° to 90°; and
2. *θ*_1_/*θ*_2_ were set as 280°/10°, 300°/30°, 315°/45°, 330°/60°, and 350°/80°, i.e., Δ*θ* was fixed at 90°.

**Table 1:** Dose criteria of RTOG 0933 protocol and additional constraints used in the current study.

	Per protocol	Variation acceptable	Deviation unacceptable
**RTOG 0933 protocol**			
Whole-brain PTV	*D*_2%_ ≤ 37.5 Gy	*D*_2%_ ≤ 40 Gy	*D*_2%_ > 40 Gy
	*D*_98%_ ≥ 25 Gy	*D*_98%_ < 25 Gy	*V*_30 Gy_ ≤ 90%
Hippocampus	*D*_100%_ ≤ 9 Gy	*D*_100%_ ≤ 10 Gy	*D*_100%_ > 10 Gy
	*D*_max_ ≤ 16 Gy	*D*_max_ ≤ 17 Gy	*D*_max_ > 17 Gy
Optic nerve and chiasm	*D*_max_ ≤ 37.5 Gy	*D*_max_ ≤ 37.5 Gy	*D*_max_ > 37.5 Gy
**Additional constraints**			
Inner ear	*D*_mean_ ≤ 15 Gy	*D*_mean_ ≤ 16 Gy	*D*_mean_ > 16 Gy
Lens	*D*_max_ ≤ 9 Gy	*D*_max_ ≤ 10 Gy	*D*_max_ > 10 Gy
Eye	*D*_mean_ ≤ 11 Gy	*D*_mean_ ≤ 12 Gy	*D*_mean_ > 12 Gy

For each patient, all plans were optimized with identical dosimetric constraints and normalized to 92% of the PTV receiving 30 Gy, while there were certain adjustments in the optimization methods for different patients to assure that all plans complied with the dose criteria.

### Treatment plan evaluation

Several parameters, including the conformity index (*CI*), homogeneity index (*HI*), MUs, and dose-volume histogram (DVH) parameters of OARs, were selected for the plan evaluation with respect to the collimator setting.

The *CI* and *HI* were defined as^26,27^

1 $$ {\text{CI}} = \frac{{{\text{TV}}_{{{\text{PIV}}}}^{{2}} }}{{{\text{TV}} \times {\text{PIV}}}} $$

and

2 $$ {\text{HI}} = \frac{{ {\text{D}}_{{{\text{2\% }}}} - {\text{D}}_{{{\text{98\% }}}} }}{{{\text{D}}_{{{\text{50\% }}}} }}{,} $$

where *TV* is the target volume, *PIV* is the prescription isodose volume, *TV*_*PIV*_ is the target volume within the prescription isodose volume, and *D*_n%_ represents the dose delivered to n% of the target volume. *CI* closer to 1 indicates better dose conformity. *HI* closer to 0 indicates better dose homogeneity.

The evaluated dosimetric parameters of OAR included the minimum, maximum, and mean doses (*D*_100%_, *D*_max_, and *D*_mean_, respectively) of the hippocampus; the *D*_mean_ of the inner ear and eye; and the *D*_max_ of the optical nerve and lens.

### Quality assurance

QA was performed for Δ*θ* = 90° plans by using local gamma-index analysis with 3%/3, 3%/2, and 2%/2 mm criteria^28^, using an a-Si 1200 Electronic Portal Imaging Device with an active area of 40 cm × 40 cm and a pixel number of 1190 × 1190. Dose images were acquired for each arc with a source-to-detector distance of 100 cm and later retrieved to the Portal Dosimetry module of the Eclipse TPS. The Portal Dose Image Prediction version 13.6.23 algorithm was used to calculate the predicted dose images.

### Statistical analysis

Friedman test was first carried out for the above parameters within each set of plans, i.e., with different Δ*θ* and same Δ*θ* (= 90°), to investigate if Δ*θ* and the specific angle of each arc could affect the plan quality of dual-arc VMAT. Wilcoxon signed-rank test was performed for multiple comparisons between the Δ*θ* = 90° and Δ*θ* < 90° plans. The comparisons were conducted in two groups, that is, (A) between plans with same *θ*_1_ but different *θ*_2_ and (B) between those with same *θ*_2_ but different *θ*_1_. The obtained *p*-values were evaluated with Bonferroni correction.

The above two tests were also used for the gamma-index passing rate evaluation in the plan QA for Δ*θ* = 90° plans. All statistical analyses were performed using IBM SPSS Statistics version 26.0 software (IBM Corporation, Armonk, NY). *p* < 0.05 was defined as statistically significant.

### Ethics approval and consent to participate

All experimental protocols were approved by the Ethics Committee of the First Hospital of Jilin University. The Ethics Committee of the First Hospital of Jilin University waived the need for informed consent. All research was performed in accordance with relevant guidelines and regulations.

## Results

**Figure 1:**
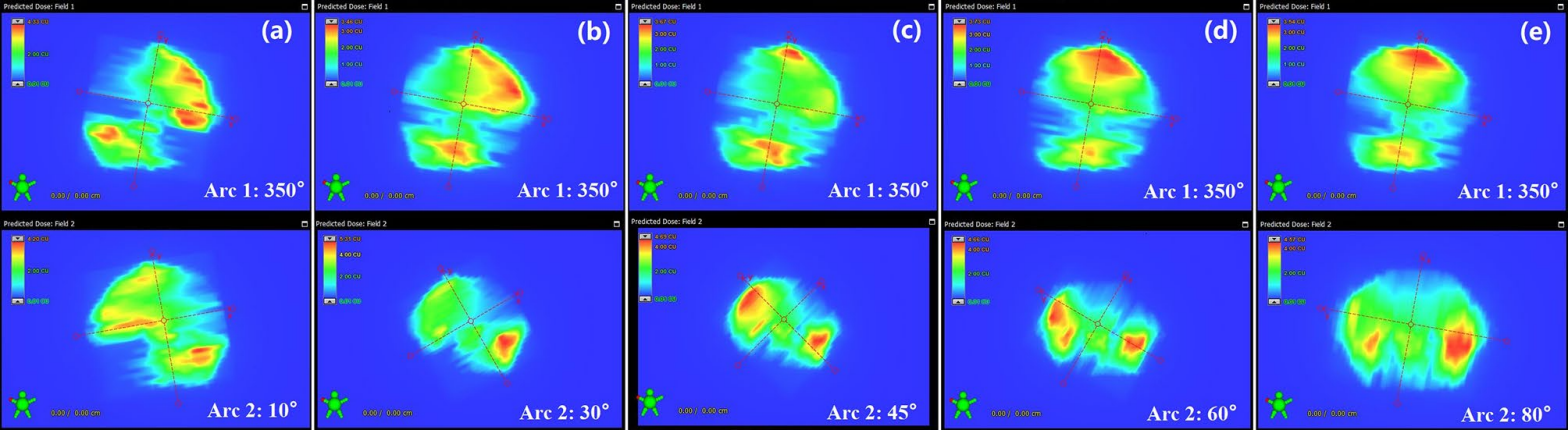
Predicted dose distribution for each arc of dual-arc VMAT plans in coronal view with collimator settings of (**a**) 350°/10°, (**b**) 350°/30°, (**c**) 350°/45°, (**d**) 350°/60°, and (**e**) 350°/80° for a typical patient.

Table 2 shows the evaluated parameters and their corresponding Friedman test results for the VMAT plans with increasing Δ*θ*. Figure 1 presents the predicted dose distribution of each arc in these plans for a typical patient. The results for plans with the same Δ*θ* (= 90°) are shown in Table 3. Supplementary Fig. S1 presents the calculated dose distribution in one 350°/80° plan for a typical patient and the corresponding DVH compared with that in Δ*θ* < 90° plans.

**Table 2:** Dosimetric parameters and MUs of VMAT plans with intersection angle of two collimators (Δ*θ*) changing from 20° to 90°, along with their Friedman test results.

	Parameter	Result for VMAT with collimator angles *θ*_1_/*θ*_2_^a^					*p-*value
		350°/10°	350°/30°	350°/45°	350°/60°	350°/80°	
PTV	CI	0.848 ± 0.009	0.853 ± 0.007	0.856 ± 0.007	0.858 ± 0.004	0.857 ± 0.008	0.002*
	*HI*	0.259 ± 0.023	0.212 ± 0.016	0.202 ± 0.016	0.199 ± 0.012	0.194 ± 0.019	< 0.001*
Hippocampus	*D*_100%_ (cGy)	929.7 ± 40.1	891.1 ± 32.8	884.2 ± 25.1	880.5 ± 31.7	878.8 ± 29.2	< 0.001*
	*D*_max_ (cGy)	1507.2 ± 105.6	1433.0 ± 77.7	1422.0 ± 65.7	1434.2 ± 76.7	1412.7 ± 79.3	< 0.001*
	*D*_mean_ (cGy)	1103.3 ± 43.3	1056.5 ± 31.0	1053.8 ± 27.6	1049.6 ± 29.7	1043.3 ± 30.3	< 0.001*
Inner ear	*D*_mean_ (cGy)	1437.6 ± 40.3	1433.0 ± 20.1	1429.5 ± 30.0	1427.4 ± 29.8	1427.3 ± 29.1	0.008*
Lens	*D*_max_ (cGy)	705.3 ± 29.3	697.2 ± 33.3	705.5 ± 32.6	703.6 ± 24.7	702.4 ± 38.1	0.699
Optical nerve	*D*_max_ (cGy)	3143.5 ± 156.3	3106.8 ± 118.2	3175.1 ± 40.4	3123.6 ± 103.1	3087.3 ± 127.0	0.053
Eye	*D*_mean_ (cGy)	970.9 ± 37.5	943.2 ± 18.3	934.0 ± 17.6	930.5 ± 11.6	921.0 ± 20.9	< 0.001*
Monitor Unit	MU	1073.6 ± 118.8	929.5 ± 44.6	908.7 ± 33.9	900.6 ± 28.5	899.5 ± 28.2	< 0.001*

^a^*θ*_1_ and *θ*_2_ indicate the collimator angles of arc 1 and arc 2, respectively.

*Statistically significant (*p* < 0.05).

**Table 3:** Dosimetric parameters and MUs of Δ*θ* = 90° plans, along with their Friedman test results.

	Parameter	Result for VMAT with collimator angles *θ*_1_/*θ*_2_^a^					*p*-value
		280°/10°	300°/30°	315°/45°	330°/60°	350°/80°	
PTV	CI	0.859 ± 0.006	0.859 ± 0.008	0.858 ± 0.007	0.855 ± 0.007	0.857 ± 0.008	0.501
	*HI*	0.189 ± 0.013	0.193 ± 0.013	0.189 ± 0.013	0.194 ± 0.011	0.194 ± 0.019	0.289
Hippocampus	*D*_100%_ (cGy)	879.4 ± 34.5	873.5 ± 27.1	872.2 ± 28.2	869.8 ± 31.3	878.8 ± 29.2	0.112
	*D*_max_ (cGy)	1415.1 ± 71.1	1432.9 ± 69.6	1411.6 ± 81.8	1415.8 ± 65.5	1412.7 ± 79.3	0.074
	*D*_mean_ (cGy)	1041.7 ± 29.8	1045.4 ± 27.5	1039.6 ± 31.0	1041.6 ± 30.8	1043.3 ± 30.3	0.412
Inner ear	*D*_mean_ (cGy)	1411.0 ± 56.0	1412.6 ± 31.1	1407.8 ± 38.1	1403.5 ± 49.4	1427.3 ± 29.1	0.106
Lens	*D*_max_ (cGy)	669.3 ± 26.1	684.6 ± 20.4	683.9 ± 24.5	698.5 ± 27.8	702.4 ± 38.1	0.001*
Optical nerve	*D*_max_ (cGy)	3106.7 ± 87.2	3180.2 ± 55.6	3180.7 ± 96.1	3171.5 ± 63.8	3087.3 ± 127.0	0.001*
Eye	*D*_mean_ (cGy)	919.4 ± 22.8	922.8 ± 13.9	918.2 ± 21.0	925.0 ± 9.4	921.0 ± 20.9	0.421
Monitor unit	MU	905.2 ± 37.5	878.4 ± 44.4	878.5 ± 36.7	881.3 ± 36.7	899.5 ± 28.2	0.003*

^a^*θ*_1_ and *θ*_2_ indicate the collimator angles of arc 1 and arc 2, respectively.

*Statistically significant (*p* < 0.05).

### Plan quality with increasing Δ*θ*

As shown in Table 2, Δ*θ* showed a significant effect on the CI, HI, MUs, and dosimetric parameters of the hippocampus (*D*_100%_, *D*_max_, and *D*_mean_), inner ear (*D*_mean_), and eye (*D*_mean_), whereas no significance was found in the *D*_max_ of lens and optical nerve.

Among the five collimator settings, the 350°/80° (Δ*θ* = 90°) plans exhibited the best HI, MUs, and dosimetric parameters of the hippocampus, inner ear, and eye and the second-best CI. In the 350°/80° plans compared with the 350°/10° ones, the CI and HI were improved by 1.1% (*p* = 0.007) and 25.2% (*p* < 0.001), respectively; MUs were reduced by 16.2% (*p* < 0.001); the *D*_100%_, *D*_max_, and *D*_mean_ to the hippocampus were reduced by 5.5% (*p* < 0.001), 6.3% (*p* < 0.001), and 5.4% (*p* < 0.001), respectively; the *D*_mean_ to the inner ear was reduced by 0.7% (*p* = 0.039); and the *D*_mean_ to the eye was reduced by 5.1% (*p* < 0.001).

**Figure 2:**
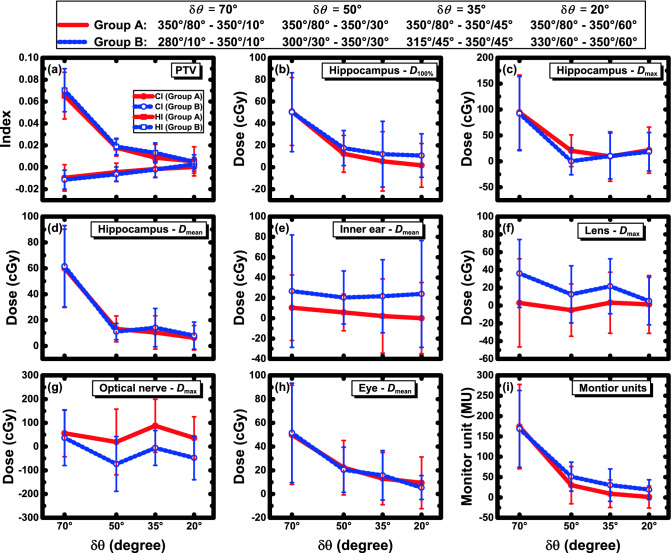
Variation of parameter differences between Δ*θ* < 90° plans and Δ*θ* = 90° plans with respect to δ*θ* (= 90° − Δ*θ*). Group A shows the differences between plans with the same arc 1 collimator, indicated by the filled symbols and solid lines. Group B shows the differences between plans with the same arc 2 collimator, indicated by the open symbols with dashed lines.

For further investigation of the relation between the plan quality and Δ*θ*, the parameter values in the Δ*θ* = 90° plans minus those in the Δ*θ* < 90° plans are shown in Fig. 2 in two groups: group A, plans with the same *θ*_1_ indicated by the solid lines; and group B, plans with the same *θ*_2_ indicated by the dashed lines. The difference in the plan Δ*θ* was denoted by δ*θ* = 90° − Δ*θ* (< 90°).

With decreasing δ*θ*, i.e., Δ*θ* increasing towards 90°, improvements were clearly demonstrated for the CI, HI, MUs, and dosimetric parameters of the hippocampus and eye. A high degree of similarity was observed between groups A and B. One-tailed Wilcoxon signed-rank tests were performed for the comparisons within groups A and B, and the results are shown in Supplementary Tables S1 and S2, respectively. Compared with the Δ*θ* < 90° plans, the Δ*θ* = 90° plans generally showed significantly better or similar results depending on δ*θ*.

### Plan quality with same Δ*θ* (90°) and different *θ*_1_/*θ*_2_

As shown in Table 3, *θ*_1_/*θ*_2_ only showed a significant correlation with the MUs and maximum doses to the lens and optical nerve. The 300°/30°, 315°/45°, and 330°/60° plans required higher MUs but reduced *D*_max_ to the optical nerve compared with the 280°/10° and 350°/80° plans. As to the *D*_max_ of lens, the 280°/10°, 300°/30°, and 315°/45° plans exhibited better results than the 330°/60° and 350°/80° plans. In general, the difference in plan quality was relatively trivial.

### Quality assurance for Δ*θ* = 90° plans

The QA results for the Δ*θ* = 90° plans are displayed in Table 4. The passing rate was statistically affected by the collimator setting, and for all gamma criteria, the 280°/10° and 350°/80° plans had significantly lower passing rates than the 300°/30°, 315°/45°, and 330°/60° plans (*p* < 0.005 for Wilcoxon signed-rank tests). The passing rates of each arc in the 280°/10° and 350°/80° plans are shown in Table 5. The results for 280° and 80° were generally similar and lower than those for 350° and 10°.

**Table 4:** Passing rates of Δ*θ* = 90° plans using 3%/3, 3%/2, and 2%/2 mm gamma criteria, along with their Friedman test results.

Gamma criteria	Passing rate for VMAT with collimator angles *θ*_1_/*θ*_2_ (%)					*p*-value
	280°/10°	300°/30°	315°/45°	330°/60°	350°/80°	
3%/3 mm	97.6 ± 0.6	98.3 ± 0.6	98.4 ± 0.5	98.5 ± 0.5	97.6 ± 0.6	< 0.001*
3%/2 mm	95.4 ± 1.0	96.9 ± 1.0	97.1 ± 0.8	97.2 ± 0.8	95.5 ± 1.1	< 0.001*
2%/2 mm	90.4 ± 2.2	92.7 ± 2.1	92.9 ± 1.8	93.1 ± 1.9	90.4 ± 2.2	< 0.001*

*Statistically significant (*p* < 0.05).

**Table 5:** Passing rates of each arc in 280°/10° and 350°/80° plans using 3%/3, 3%/2, and 2%/2 mm gamma criteria.

Gamma criteria	Passing rate for 280°/10° (%)		Passing rate for 350°/80° (%)	
	Arc 1 (280°)	Arc 2 (10°)	Arc 1 (350°)	Arc 2 (80°)
3%/3 mm	98.7 ± 0.6	99.1 ± 0.3	99.3 ± 0.3	97.8 ± 1.4
3%/2 mm	95.6 ± 2.2	97.6 ± 0.7	98.0 ± 0.6	95.0 ± 2.6
2%/2 mm	92.2 ± 2.8	96.1 ± 1.1	96.7 ± 1.0	91.6 ± 3.1

## Discussion

Considering the positive results demonstrated in the RTOG 0933 trail, HIS-WBRT has gradually become a common practice for the treatment of brain metastases and prophylactic cranial irradiation^7–9,29–31^. The plan quality is especially important, and choosing the collimator angle is a vital part of the plan optimization to achieve an ideal dose distribution due to the complexity of HIS-WBRT. In the present study, the collimator angle had a significant effect on the dose distribution quality and MUs, in agreement with the previous studies. Zhang et al.^32^ developed a collimator trajectory selection method for VMAT, and an enhanced dose distribution could be achieved by aligning the collimator with the target shape. Several studies showed that the VMAT optimization involving sectional optimization of collimator angle could provide delivery efficiency and dosimetric improvements^33,34^. Recent studies on the dynamic collimator rotation approach have shown positive results on improving the plan quality^24,35–37^.

Table 2 shows that among the five sets of plans with different Δ*θ*, the 350°/80° (Δ*θ* = 90°) plans had the best overall dosimetric performance and MUs. Figure 2 demonstrates the differences between the Δ*θ* = 90° and Δ*θ* < 90° plans with respect to δ*θ*. A visible similarity was present between groups A and B in the improvement of CI, HI, MUs, and doses to the hippocampus and eye with decreasing δ*θ*, indicating that with Δ*θ* increasing towards 90°, the plan quality could be gradually improved.

The five sets of Δ*θ* = 90° plans were compared to investigate the possible effect from the *θ*_1_/*θ*_2_ combination with the same Δ*θ*, and the results are shown in Table 3. Thus, when the intersection Δ*θ* was the same, most parameters showed no significant correlation with *θ*_1_/*θ*_2_ combinations, except the MUs and maximum doses to lens and optical nerve. Considering the behavior of all parameters, the effect of Δ*θ* was much greater than that of the specific angle of each arc.

The collimator angle should allow the MLC aperture to encompass the PTV while avoiding the sparing region. For the near-spherical target volume in HIS-WBRT, with the avoidance of two hippocampi, which exist bilaterally in the middle of the brain, the plan complexity could be considerably increased compared with conventional WBRT. Many studies proposed that high plan complexity could affect the accuracy of dose calculation and beam delivery^38–40^. The predicted dose distribution shown in Fig. 1 indicated that regardless of the collimator setting, the plan optimization tended to form mutually orthogonal dose distributions in two arcs, and the integrated dose distribution could then conform with the complex-shape target volume of HIS-WBRT. With the Δ*θ* = 90° plans, i.e., the MLC orientations of two arcs perpendicular to each other, the motion complexity of MLC could be potentially reduced compared with the Δ*θ* < 90° plans, which could be part of the reason for the improved dose distribution quality of Δ*θ* = 90° plans.

Previous studies on the effect of collimator angle on VMAT plan quality proposed various optimal settings depending on the tumor site and shape^12,17,41^. For vertebrae metastases, Mancosu et al. suggested a collimator angle of around 90° to align the MLC leaf motion with the spinal cord^41^. For prostate cancer, Li et al. suggested an angle of 45° for the optimal dose distribution plan complexity^17^. For nasopharynx cancer, Otto found that 45° was preferable in most cases^12^. In the present study, the optimal collimator setting is affected by the complex-shaped target of HIS-WBRT and thus different from the above findings. For the dual-arc VMAT technique adopted for HIS-WBRT, the optimal plan quality was acquired with Δ*θ* = 90°, while the effect from the specific setting of each arc was relatively insignificant.

For the Δ*θ* = 90° plans with different collimator angle combinations, the gamma-index analysis results shown in Tables 4 and 5 indicated that the passing rates could be affected by the specific angle of each arc. Although the passing rate with the collimator closer to 0° showed preferable results in this study, it should be noted that the QA result could be associated with many factors, such as the dosimetric leaf gap, tongue-and-groove effect, interleaf leakage, and intra- and inter-leaf transmission settings of MLC^42–45^. For the PDIP algorithm in the Eclipse TPS, these MLC configuration parameters are constant for all collimator and gantry angles, but in practice, they may vary for different situations. The leaf position accuracy could be affected by the gantry rotation with different collimator angles due to gravitational effects imposed on the leaf carriage system^45^. The varying MLC parameters could yield a significant dose error, especially for highly modulated plans. Therefore, with regard to the plan QA for other institutions, the preferable result may vary.

## Conclusions

This study demonstrated the role of collimator settings in dual-arc VMAT planning for HIS-WBRT. The intersection angle between two collimator settings, Δ*θ*, showed a significant influence on the plan quality. With Δ*θ* approaching 90°, a nearly continuous improvement was observed in the dose distributions and MUs. However, with the same Δ*θ* = 90°, the effect of specific collimator angle of each arc was relatively trivial. The QA results indicated that the 300°/30°, 315°/45°, and 330°/60° plans had a better delivery accuracy than the 280°/10° and 350°/80° plans.

## Supplementary Information

Supplementary Information.

## Data Availability

Research data analyzed in this study are included as supplementary materials. Other data that support the findings of this study are available from the corresponding author upon reasonable request.
