# Supplementary material for: Optimization of collimator angles in dual-arc volumetric modulated arc therapy planning for whole-brain radiotherapy with hippocampus and inner ear sparing
Source: Sci Rep. 2021 Sep 24;11:19035. doi: 10.1038/s41598-021-98530-7 (PMC8463591; doi:10.1038/s41598-021-98530-7)
Supplement: Supplementary file 1 — Supplementary Information. [file 41598_2021_98530_MOESM1_ESM.docx]

**Supplementary Materials**


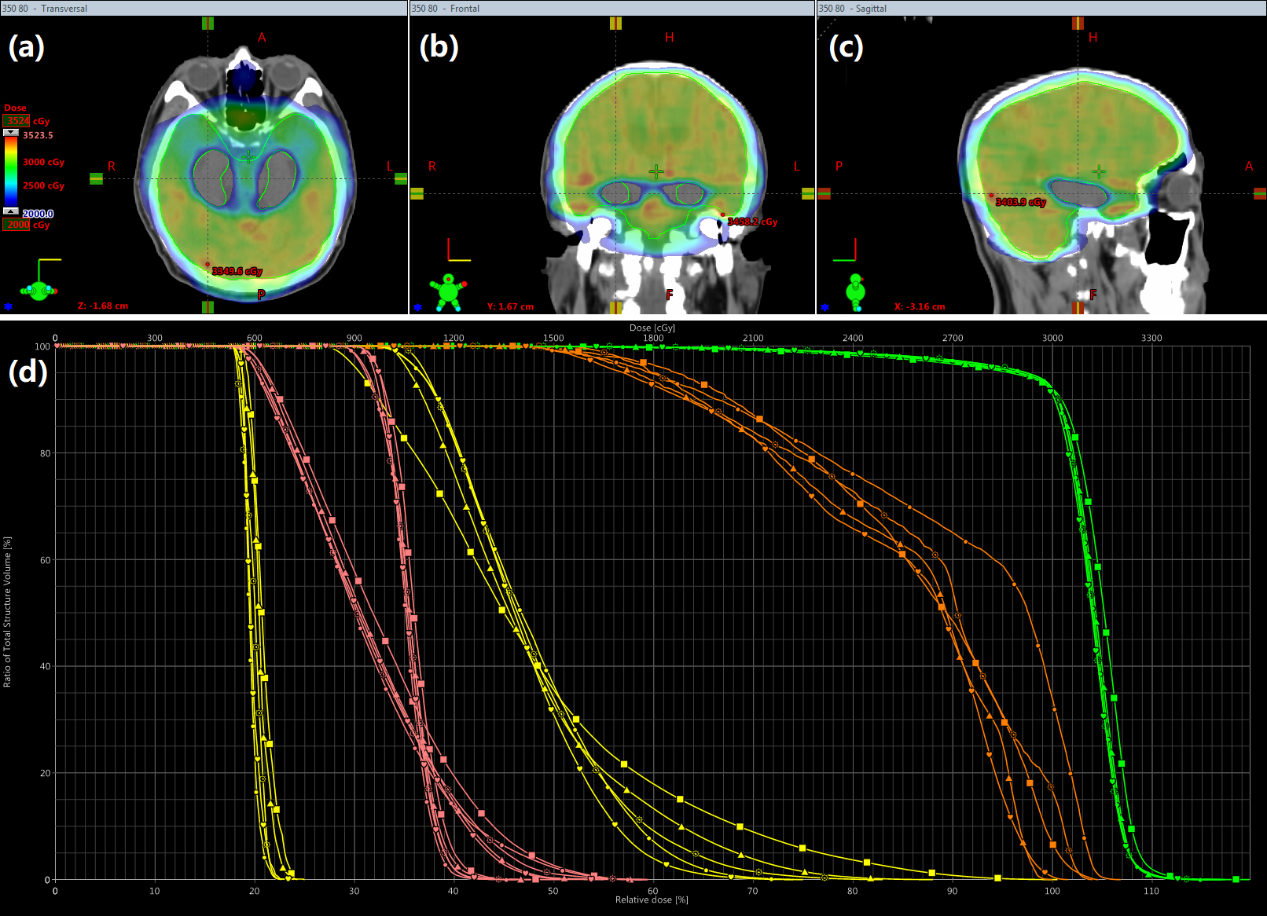


**Figure S1.** Dose distribution for a typical 350°/80° HIS-WBRT VMAT plan in (a) transversal, (b) frontal, and (c) sagittal views. (d): Dose volume histograms of VMAT plans with different Δ*θ* (350°/10°, 350°/30°, 350°/45°, 350°/60°, and 350°/80°).

**Table S1.** Wilcoxon singed-rank test results between Δθ < 90° plans and Δθ = 90° plans with the same arc 1 collimator angle.

| **Parameter** | | **Wilcoxon signed-rank test *p*-value** | | | |
| --- | --- | --- | --- | --- | --- |
|  |  | **350°/80° - 350°/10°** | **350°/80° - 350°/30°** | **350°/80° - 350°/45°** | **350°/80° - 350°/60°** |
| **PTV** | **CI** | 0.007** | 0.029* | 0.249 | 0.473 |
|  | **HI** | <0.001** | <0.001** | 0.009** | 0.122 |
| **Hippocampus** | **D_100%_ (cGy)** | <0.001** | 0.015* | 0.243 | 0.339 |
|  | **D_max_ (cGy)** | <0.001** | 0.014* | 0.393 | 0.042* |
|  | **D_mean_ (cGy)** | <0.001** | 0.001** | 0.008** | 0.018* |
| **Inner ears** | **D_mean_ (cGy)** | 0.039* | 0.228 | 0.467 | 0.395 |
| **Lenses** | **D_max_ (cGy)** | 0.304 | 0.400 | 0.400 | 0.393 |
| **Optical nerves** | **D_max_ (cGy)** | 0.013* | 0.446 | 0.011** | 0.098 |
| **Eyes** | **D_mean_ (cGy)** | <0.001** | 0.003** | 0.016* | 0.081 |
| **Monitor Unit** | **MU** | <0.001** | 0.011** | 0.183 | 0.111 |

* Statistically significant without a Bonferroni correction (*p* < 0.05).

** Statistically significant with a Bonferroni correction (*p* < 0.0125).

**Table S2.** Wilcoxon singed-rank test results between Δθ < 90° plans and Δθ = 90° plans with the same arc 2 collimator angle.

| **Parameter** | | **Wilcoxon signed-rank test *p*-value** | | | |
| --- | --- | --- | --- | --- | --- |
|  |  | **280°/10° - 350°/10°** | **300°/30° - 350°/30°** | **315°/45° - 350°/45°** | **330°/60° - 350°/60°** |
| **PTV** | **CI** | <0.001** | <0.001** | 0.122 | 0.064 |
|  | **HI** | <0.001** | <0.001** | <0.001** | 0.005** |
| **Hippocampus** | **D_100%_ (cGy)** | <0.001** | 0.002** | 0.058 | 0.029* |
|  | **D_max_ (cGy)** | <0.001** | 0.202 | 0.235 | 0.029* |
|  | **D_mean_ (cGy)** | <0.001** | <0.001** | 0.003** | 0.012** |
| **Inner ears** | **D_mean_ (cGy)** | 0.009** | 0.011** | 0.013* | 0.071 |
| **Lenses** | **D_max_ (cGy)** | 0.003** | 0.073 | 0.010** | 0.266 |
| **Optical nerves** | **D_max_ (cGy)** | 0.193 | 0.061 | 0.500 | 0.056 |
| **Eyes** | **D_mean_ (cGy)** | <0.001** | <0.001** | 0.004** | 0.040* |
| **Monitor Unit** | **MU** | <0.001** | <0.001** | <0.001** | 0.002** |

* Statistically significant without a Bonferroni correction (*p* < 0.05).

** Statistically significant with a Bonferroni correction (*p* < 0.0125).
